# Cancer information needs according to cancer type: A content analysis of data from Japan's largest cancer information website

**DOI:** 10.1016/j.pmedr.2018.10.014

**Published:** 2018-10-22

**Authors:** Tsuyoshi Okuhara, Hirono Ishikawa, Akiko Urakubo, Masayo Hayakawa, Chikako Yamaki, Tomoko Takayama, Takahiro Kiuchi

**Affiliations:** a Department of Health Communication, School of Public Health, The University of Tokyo, 7-3-1 Hongo, Bunkyo-ku, Tokyo 113-8655, Japan; b School of Public Health, Teikyo University School of Medicine, 2-11-1 Kaga, Itabashi-ku, Tokyo 173-8605, Japan; c Division of Cancer Information Service, Center for Cancer Control and Information Services, National Cancer Center, 5-1-1, Tsukiji, Chuo-ku, Tokyo 104-0045, Japan

**Keywords:** Cancer, Neoplasms, Consumer health information, Information services, Information dissemination, Information seeking behavior, Cancer patients, Cancer survivors, Health communication

## Abstract

The provision of information about cancer is an important aspect of cancer care. Cancer information provided online is expected to meet the needs of information seekers. Whether information needs vary according to tumor site is largely unknown. We aimed to examine similarities and differences in informational needs by cancer type. Data were collected using a questionnaire administered on Japan's largest cancer information website, “Ganjoho service”. A total of 2782 free descriptive responses in the period from April 2012 to December 2017 were analyzed using text-mining software. We identified the top 10 informational need contents, in order of appearance frequency, for eight tumor sites: gastric, colorectal, esophageal, lung, pancreatic, breast, cervical, and prostate cancer. Frequent information needs common to all tumor sites included symptoms, disease stages, treatments, chance of cure, recovery, metastasis, and recurrence. A need for information about diet, pain, side effects of treatments, complementary and alternative medicine was frequent for some tumor sites. Tumor site-specific information should include the following, according to cancer type: information of scirrhous carcinoma for gastric cancer; unusual feces for colorectal cancer; lung X-ray images for lung cancer; early detection for pancreatic cancer; adenocarcinoma, sexual activity, pregnancy, and childbirth for cervical cancer; breast conservation or reconstruction and triple negative cancer for breast cancer; test values and diagnosis and urinary problems for prostate cancer; and hormone therapy for breast and prostate cancer. Cancer information provided online should meet these frequent informational needs, considering similarities and differences of the information required according to tumor site.

## Highlights

• We examined similarities and differences in cancer information needs by tumor site.
• Common needs are symptoms, stages, treatment, cure, recovery, metastasis, recurrence.
• Site-specific needs include diet, pain, side effects, early detection, test values.
• Cancer information should be provided to meet frequent needs, by tumor site.

## 1 Introduction

The internet is increasingly used as a key source of cancer information among cancer patients, survivors, and care givers (James et al., 2007; Dolce, 2011; Selsky et al., 2013). Approximately 90% of the population regularly accesses the internet in Europe, North America, and Japan (Internet World Stats, 2017). Websites are one of the main sources of cancer information in Japan (Tokyo Metropolitan Government Bureau of Social Welfare and Public Health, 2013). Providing cancer information online has several benefits for patients, survivors, and care givers. For example, the internet provides widespread and easy access to cancer information (Koch-Weser et al., 2010), and information seekers are free to consult information anonymously (Schook et al., 2014). These benefits may allow them to increase their knowledge, self-efficacy, and ability to actively participate in making health care decisions (Maddock et al., 2012).

Universal health insurance has improved equity in the health system of Japan. However, unlike in many other developed countries, the primary care doctor system is underdeveloped in Japan. Many people do not have health professionals with whom they can consult about their symptoms. Additionally, cancer patients often have difficulties understanding the doctor's explanation or asking doctors about things that they do not understand (Arora, 2003). Therefore, people who are experiencing symptoms and cancer patients often seek information on the Internet (Huang and Penson, 2008). Cancer information websites need to address the needs of such individuals.

However, many studies indicate that many cancer patients are not satisfied with the content of information they receive online (Puts et al., 2012; Goldfarb and Casillas, 2014; Warren et al., 2014; Faller et al., 2016). Improvement of online cancer information is needed to meet the needs of cancer information seekers attempting to fill gaps in information (Warren et al., 2014). Most previous studies have examined information needs based on a single tumor site (e.g., Noh et al., 2009; Henselmans et al., 2012; Van Mossel et al., 2012; Halbach et al., 2016; Kassianos et al., 2016) or have reported results on mixed tumor sites (e.g., Rutten et al., 2005; Tariman et al., 2014; Faller et al., 2016). Thus, how the need for information varies by tumor site is largely unknown (Shea–Budgell et al., 2014).

Revealing similarities in the informational needs according to tumor site will contribute to prioritizing the contents of cancer information overall. Additionally, clarifying differences in the informational needs by tumor site can also assist in improving cancer information provided to patients with specific cancers. Therefore, in the present study, we examined the most frequent cancer informational needs according to tumor site, and discussed the similarities and differences, using data collected via a questionnaire administered on Japan's largest cancer information website.

## 2 Methods

### 2.1 Study design

We conducted quantitative content analysis, a research method to quantitatively examine the presence of certain concepts within sets of text. We used a text-mining method and software (described below) to analyze a large set of text.

### 2.2 Data collection

We collected text data from “Ganjoho service” (https://ganjoho.jp), which is a website that the National Cancer Center Japan has maintained since 2006. *Gan* means cancer and *joho* means information in Japanese. “Ganjoho service” is the largest cancer information website in Japan, with an average 2.7 million views per month in 2017. Many cancer-related search terms such as “breast cancer” and “cancer treatment” return “Ganjoho service” in the top three search results of the two most popular search engines in Japan, Google Japan (www.google.co.jp) and Yahoo! Japan (www.yahoo.co.jp) (accessed on March 26, 2018). As of December 2017, “Ganjoho service” provides information about 55 kinds of cancer, with separate webpages for each cancer type. Editors of “Ganjoho service” update the contents based on clinical practice guidelines. Cancer-related clinical practice guidelines are updated once every a few years in Japan. Editors make an effort to update contents as soon as possible after the guidelines are updated. Other new information such as statistics and institution-related information is updated from time to time.

Individual webpages of “Ganjoho service” provide exhaustive information such as morbidity and mortality, symptoms, examination and diagnosis, disease stages, treatment options, side effects of treatments, chance of cure, recovery and rehabilitation, metastasis, and recurrence. To improve the information provided, all webpages have links to a questionnaire. Website visitors complete the questionnaire if they consent to the use of their responses for research purposes; an explanation of research uses of responses is provided for visitors to read prior to giving their consent. The questionnaire is used to collect information on the respondent's status (i.e., patients and survivors, family members of patients and survivors, other persons than patients and survivors and their family members) in a multiple-choice question. Additionally, information needs are queried using an anonymous free descriptive format with the question, “Please describe the information you were seeking but could not obtain.” In the present study, we defined informational needs as “the information that visitors needed but could not obtain in Ganjoho service.” In the period from April 2012 to December 2017, a total of 2794 responses were received for questionnaires linked to pages for eight tumor sites: prostate, breast, pancreatic, lung, colorectal, gastric, cervical, and esophageal cancer. These eight tumor sites represent the cancers with the most frequently completed questionnaires among all cancer types. We excluded 12 irrelevant responses (e.g., addressing tumor sites other than these eight), and we analyzed the remaining 2782 responses. The study was approved by the ethical review committee at the Graduate School of Medicine, The University of Tokyo.

### 2.3 Coding procedure

We analyzed the data using a text-mining method with KH Coder Version 2.00f software (Higuchi, 2001, Higuchi, 2012) for quantitative content analysis. KH Coder, which supports Japanese text, uses the ChaSen Morphological Analyzer and R statistical software environment. KH Coder has been successfully used in public health studies both in and outside of Japan (Goto et al., 2014; McNeill et al., 2016). We conducted the following procedures for each tumor site. We applied one paragraph as the calculation unit in cluster analysis and network analysis as follows.

First, the first author (T.O.) conducted hierarchical cluster analysis (Ward's method) to examine the appearance pattern of terms (Ward, 1963). Analysis results were presented using a dendrogram, within which lines were drawn to show clusters of terms that were close in their appearance pattern. This analysis helped with exploring how terms were used in the materials (Higuchi, 2001).

Second, the first author extracted the top 100 terms in order of the probability of appearance. He then conducted network analysis of the examined co-occurrence of frequently appearing terms (Osgood, 1959; Danowski, 1993). Analytical results were presented as the figure of a network, within which terms with a great degree of co-occurrence were linked to each other. The degree of co-occurrence was determined using the Jaccard similarity coefficient (Niwattanakul et al., 2013). This analysis helped in exploring the appearance pattern of terms, as well as the contents represented by the linked terms (Higuchi, 2001).

Third, the first author created codes and coding rules representing specific contents by combining frequently appearing co-occurring terms. In creating the codes, he also referred to typology, categories, and topics of cancer information needs that have been suggested in previous studies (Rutten et al., 2005; van Weert et al., 2013; Tariman et al., 2014; Warren et al., 2014). Additionally, he consulted other authors (A.U., M.H., C.Y., and T.T.) who were professionals of cancer information research to create as many codes as possible to exhaustively analyze frequently appearing content. Finally, we identified the top 10 codes in order of appearance frequency for each tumor site.

### 2.4 Analysis

Descriptive statistics were used to calculate and summarize the data. Percentages were calculated by dividing the number of code-fitted paragraphs by the number of total paragraphs for all respondents as a primary analysis, and for each respondent status (i.e., patients and survivors, family members of patients and survivors, and persons other than patients and survivors and their family members) as a sub-group analysis. The chi-square test was applied to assess the significance of differences in the distribution of code-fitted paragraphs between respondent statuses. Statistical significance was set at p < 0.05. The analyses were conducted using KH Coder, Version 2.00f (Higuchi, Ritsumeikan University, Kyoto, Japan).

## 3 Results

Table 1 shows the number of terms, unique terms, paragraphs, answers analyzed, and distribution of respondents. About 30%–60% of respondents were patients and survivors, and the remainder were family members of patients and others, such as individuals who were acquaintances of patients and people with subjective symptoms of cancer. In total, 23,875 terms, 7032 unique terms, and 3009 paragraphs were analyzed.

**Table 1:** Number of terms, unique terms, paragraphs, responses analyzed, and distribution of respondents.

Tumor site	Responses	Respondents (%)			Terms	Unique terms	Paragraphs
		Patients and survivors	Family members	Others^a^			
Gastric cancer	244	49	29	22	2058	724	272
Colorectal cancer	370	43	33	24	3234	954	394
Esophageal cancer	205	43	31	26	1798	610	219
Lung cancer	389	42	35	23	3331	986	432
Pancreatic cancer	407	31	39	30	3326	960	441
Breast cancer	455	63	15	22	3778	1018	502
Cervical cancer	207	53	18	29	1875	599	216
Prostate cancer	505	52	17	31	4475	1181	533
Total	2782	47	27	26	23,875	7032	3009

a Others: Individuals who are acquaintances of patients, people feeling subjective symptoms of cancer, and so on.

**Fig. 1:**
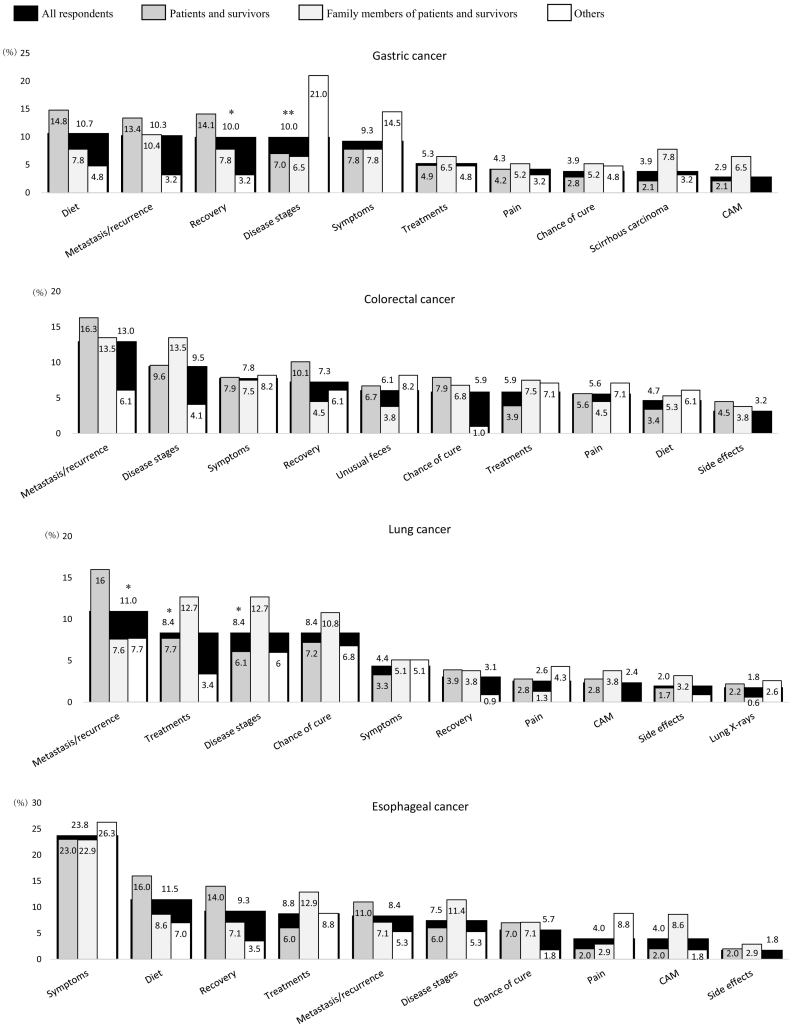

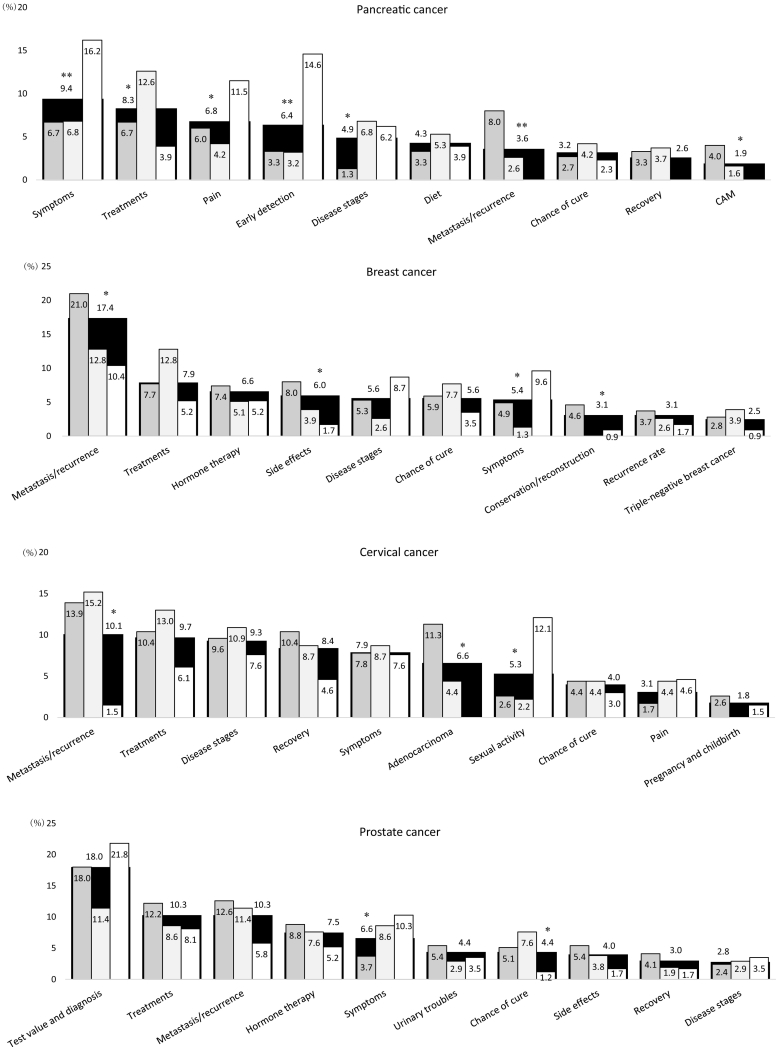
Distribution of code-fitted paragraphs in order of higher appearance frequency, by tumor site and respondents' status. *p < 0.05, **p < 0.01, chi-square test in comparison between respondents' statuses. CAM: Complementary and alternative medicine.

Table 2 shows codes that appeared frequently for all, some, or only one of the top eight tumor sites. Fig. 1 shows the distribution of code-fitted paragraphs for each tumor site and each respondent's status. The top five most frequently appearing codes were as follows, according to cancer type: diet, metastasis and recurrence, recovery, disease stages, and symptoms for gastric cancer; metastasis and recurrence, disease stages, symptoms, recovery, and unusual feces for colorectal cancer; metastasis and recurrence, treatments, disease stages, chance of cure, and symptoms for lung cancer; symptoms, diet, recovery, treatments, and metastasis and recurrence for esophageal cancer; symptoms, treatments, pain, early detection, and disease stages for pancreatic cancer; metastasis and recurrence, treatments, hormone therapy, side effects of treatment, and disease stages for breast cancer; metastasis and recurrence, treatments, disease stages, recovery, and symptoms for cervical cancer; and test values and diagnosis, treatments, metastasis and recurrence, hormone therapy, and symptoms for prostate cancer. Of the total 80 codes for the eight tumor sites, significant differences in the distribution of code-fitted paragraphs between respondents' statuses were found in 20 codes, such as recovery for gastric cancer (p < 0.05), metastasis and recurrence for lung cancer (p < 0.05), symptoms for pancreatic cancer (p < 0.01), and side effects of treatments for breast cancer (p < 0.05); see Fig. 1 for more information.

**Table 2:** Codes frequently appearing in all, some, or one of eight tumor sites.

Tumor site	Code^a^	Example of terms used in coding rules^a^
All	Symptoms	Symptom, initial symptom, subjective symptom
	Disease stages	Stage, degree of progress
	Treatments	Methods of treatment, surgical treatment
	Chance of cure	Survival rate, likelihood of cure, life expectancy
	Recovery	Recovery, postoperative care, life after hospital discharge, rehabilitation
	Metastasis and recurrence	Metastasis, recurrence
Gastric cancer^b^	Diet	Diet, food, eating and drinking, liquor, menu
	Scirrhous stomach cancer	Scirrhous
	Pain	Pain, stomachache
	Complementary and alternative medicine	Herbal medicine, immunotherapy, non-standard care
Colorectal cancer^b^	Unusual feces	Bloody stool, diarrhea, loose stool, constipation, fecal incontinence
	Pain	Pain, lower abdominal pain
	Diet	Diet, food, eating and drinking, liquor, menu
	Side effects of treatments	Side effects, adverse drug reaction
Esophageal cancer^b^	Diet	Diet, food, eating and drinking, liquor, menu
	Pain	Pain, esophagodynia
	Complementary and alternative medicine	Herbal medicine, immunotherapy, non-standard care
	Side effects of treatments	Side effects, adverse drug reaction
Lung cancer^b^	Pain	Pain, stethalgia
	Complementary and alternative medicine	Herbal medicine, immunotherapy, non-standard care
	Lung X-rays	X-rays, images
	Side effects of treatments	Side effects, adverse drug reaction
Pancreatic cancer^b^	Early detection	Early detection
	Pain	Pain, epigastralgia, backache
	Diet	Diet, food, menu
	Complementary and alternative medicine	Herbal medicine, immunotherapy, non-standard care
Breast cancer^b^	Hormone therapy	Hormone therapy
	Side effects of treatments	Side effects, adverse drug reaction
	Breast conservation/reconstruction	Breast conservation, breast reconstruction
	Triple-negative breast cancer	Triple-negative cancer
Cervical cancer^b^	Adenocarcinoma	Adenocarcinoma
	Sexual activity	Sex, intercourse, coital pain
	Pain	Pain, lower abdominal pain, low back pain
	Pregnancy and childbirth	Pregnancy, childbirth
Prostate cancer^b^	Test value and diagnosis	Test value, criteria, diagnose
	Hormone therapy	Hormone therapy
	Urinary problems	Hematuria, pollakiuria, incontinence
	Side effects of treatments	Side effects, adverse drug reaction

a Authors translated terms from Japanese to English for the purpose of this report.

b Codes are in descending order of appearance frequency.

## 4 Discussion

We identified the top 10 informational needs in order of appearance frequency for each of the eight studied tumor sites: gastric, colorectal, lung, esophageal, pancreatic, breast, cervical, and prostate cancer. Previous studies have examined cancer informational needs according to individual tumor sites (e.g., Noh et al., 2009; Henselmans et al., 2012; Van Mossel et al., 2012; Halbach et al., 2016; Kassianos et al., 2016) or mixed tumor sites (e.g., Rutten et al., 2005; Tariman et al., 2014; Faller et al., 2016). However, those studies adopted a somewhat limited view of informational needs across different tumor sites. Therefore, the informational needs that are common to different tumor sites have been unknown. Additionally, those previous studies did not compare informational needs between different tumor sites. Therefore, differences in informational needs by tumor site have also been unknown. Fig. 1 in the present study presents an extensive view of informational needs according to eight tumor sites, facilitating comparison of these information needs between tumor sites. This extensive point of view as well as the comparisons made represent important contributions of this study to cancer information research. Below, we discuss similarities in the informational needs according to different tumor sites and characteristics of the informational needs by each tumor site.

### 4.1 Similarities in the informational needs by tumor site

Symptoms, disease stages, treatments, chance of cure, recovery, metastasis, and recurrence were the most frequently appearing information needs common to eight tumor sites: gastric, colorectal, esophageal, lung, pancreatic, breast, cervical, and prostate cancers. This result is nearly consistent with those of previous studies examining cancer information needs for mixed tumor sites (Rutten et al., 2005; Shea–Budgell et al., 2014; van Weert et al., 2013; Tariman et al., 2014; Warren et al., 2014; Tan et al., 2015; Faller et al., 2016). In the present study, we found that patients, survivors, and others may seek these types of information regardless of the type of cancer. In general, online cancer information is expected to provide such information. Information needs regarding the side effects of treatment frequently appeared for colorectal, lung, esophageal, breast, and prostate cancers. Information needs regarding complementary and alternative medicine were frequent for gastric, lung, esophageal, and pancreatic cancers. Additionally, informational needs regarding pain were frequent for gastric, colorectal, lung, esophageal, pancreatic, and cervical cancers. These contents should be considered highly sought information across those tumor sites, and cancer-related websites should provide such information, based on scientific evidence.

### 4.2 Differences in the informational needs by tumor site

The characteristics of informational needs for each tumor site were found. Information provided online should aim to meet these needs for each type of cancer addressed below.

#### 4.2.1 Gastric cancer

The reason that information about metastasis and recurrence was highly sought may be due to the 5-year survival rate of gastric cancer in Japan, which is relatively high at 64.6% of cases diagnosed in 2006–2008 (National Cancer Center Japan, 2016). Survivors of gastric cancer may worry about the possibility of metastasis and recurrence after treatment. High informational needs regarding metastasis and recurrence of gastric cancer have also been reported in a previous study, showing that patients with gastric cancer rate information about metastasis and recurrence as having the greatest importance (McNair et al., 2013). With respect to informational needs of diet and recovery, our result may indicate that patients and survivors have anxieties and questions about daily self-care after treatment, including diet, because appropriate dietary management is important after treatment for gastric cancer (Doyle et al., 2006). The need for information regarding scirrhous carcinoma was specific to gastric cancer.

#### 4.2.2 Colorectal cancer

Our study results are nearly consistent with those of a previous study that examined colorectal cancer information needs in a literature review (Van Mossel et al., 2012), showing that informational content such as metastasis and recurrence, disease stages, and chance of cure were highly sought among patients. The reason for high informational requirements regarding metastasis and recurrence may be that the 5-year survival rate of colorectal cancer in Japan is relatively high at 71.1% of cases diagnosed in 2006–2008 (National Cancer Center Japan, 2016). Information was sought about unusual feces only for colorectal cancer. Information about diet was also sought, which is consistent with a previous study showing that difficulty with diet and malnutrition are prevalent, and therefore, of high interest among surgical patients with colorectal cancer (Garth et al., 2010).

#### 4.2.3 Lung cancer

Our study result is somewhat consistent with those of a previous study showing that patients with lung cancer rated symptoms and treatments as highly important (Jacobs-Lawson et al., 2009). The reason for high information needs regarding treatments, disease stages, and chance of cure may be that lung cancer is the most common cancer in Japan in terms of the number of deaths, with 73,838 lung cancer deaths in 2016 (National Cancer Center Japan, 2018). Information sought about lung X-rays was specific to lung cancer.

#### 4.2.4 Esophageal cancer

The appearance of information needs about symptoms was remarkably frequent for esophageal cancer. Because swallowing is an integral part of daily life, people may tend to notice subjective symptoms that may be suggestive of cancer of the esophagus more often than for other sites. This result suggests that information about symptoms of esophageal cancer may be valuable. Previous studies have targeted information needs following the diagnosis of and treatment for esophageal cancer; however, the need for information regarding symptoms before diagnosis was not revealed in those studies (Andreassen et al., 2005; Andreassen et al., 2007; Henselmans et al., 2012; Smets et al., 2012). Information needs about diet and recovery followed those for symptoms. This result may indicate that patients and survivors have anxieties and questions about daily self-care after treatment, including diet, because appropriate management of the diet is needed after treatment for esophageal cancer (Berretta et al., 2012).

#### 4.2.5 Pancreatic cancer

Pancreatic cancer is less symptomatic and screening and early detection more difficult than other cancers. Symptoms can include pain in the back and lower back (Vincent et al., 2011). Additionally, pancreatic cancer is harder to treat than other cancers (Vincent et al., 2011). The reason for the high informational needs of symptoms, pain, early detection, and treatment may be these characteristics of pancreatic cancer. Our results may indicate that individuals are highly interested in early detection of pancreatic cancer, including noticing subjective symptoms such as pain, and that patients with pancreatic cancer consider information about treatments to be highly important. Informational needs regarding diet were also frequent for this tumor site. This may be because patients are prone to indigestion and diarrhea after treatment for pancreatic cancer (Muniraj et al., 2013).

#### 4.2.6 Breast cancer

Information needed about metastasis and recurrence was remarkably frequent. This may be because the 5-year survival rate of breast cancer is high in Japan at 91.1% of cases diagnosed in 2006–2008 (National Cancer Center Japan, 2016). Accordingly, many survivors are anxious about metastasis and recurrence (Ito et al., 2014). Informational needs regarding treatments, hormone therapy, and side effects of treatment were the next most frequent for breast cancer. This high interest in content related to treatment is consistent with previous studies examining the informational needs of patients with breast cancer (Li et al., 2011; Valero-Aguilera et al., 2014; Miyashita et al., 2015; Halbach et al., 2016). Information needed regarding hormone therapy, breast conservation or reconstruction, and triple negative cancer was specific to breast cancer.

#### 4.2.7 Cervical cancer

Our study result is nearly consistent with the results of a previous study examining the information needs of patients with cervical cancer at diagnosis, during treatment, and after treatment (Noh et al., 2009), which showed that information about metastasis and recurrence, treatments, and disease stages were highly sought. Information needs about adenocarcinoma, sexual activity, and pregnancy and childbirth were specific to cervical cancer. Among these, sexual issues are often overlooked in communication between health care professionals and patients. Therefore, patients and survivors may seek information about sexual issues related to cervical cancer on the internet.

#### 4.2.8 Prostate cancer

The most frequent information needs for prostate cancer were regarding test value and diagnosis, and this content was specific to prostate cancer. The information sought was related to questions about diagnosis based on PSA levels and Gleason score. This result may indicate that individuals who have undergone prostate cancer screening and prostate cancer patients are highly interested in how to evaluate the results of their tests. Tests and diagnosis have also been reported as important topics in previous studies examining the information needs of patients with prostate cancer (Feldman-Stewart et al., 2010; Bernat et al., 2016). Information needed about hormone therapy and urinary problems were specific to prostate cancer. Previous studies have also reported urinary problems as being important informational needs for this type of cancer (Echlin and Rees, 2002; Feldman-Stewart et al., 2010; Bernat et al., 2016). Other frequently appearing information needs included treatments, metastasis and recurrence, and symptoms, which are also consistent with previous studies (Echlin and Rees, 2002; Feldman-Stewart et al., 2010; Bernat et al., 2016).

### 4.3 Limitations

First, the creation of codes and coding rules may have reflected author bias, although we systematically analyzed textual data using a text-mining method. Second, this study was not a survey querying participants about their information needs but rather involved analysis of the content of responses to a questionnaire on a cancer information website. Third, because people cannot recall information that they do not know, the study results do not reflect informational needs that respondents do not know that they have. Fourth, when comparing the distribution of code-fitted paragraphs between respondents' statuses, the number of paragraphs analyzed was small owing to stratification. Because p-values depend on the sample size of the data analyzed, it is unclear to what extent the significant differences between respondents' statuses in the present study are generalizable to other populations. Finally, the results of the present study may have been related to the cancer statistics in Japan.

## 5 Conclusions

Online cancer information materials, such as the websites of cancer-related institutes, are generally expected to provide content regarding symptoms, disease stages, treatments, chance of cure, recovery, metastasis, and recurrence. When they provide information to patients with specific cancers, informational materials are expected to meet the information needs for different types of cancer that were showed in the present study. Making efforts to appropriately provide cancer information will lead to great benefits such as increased knowledge and coping ability of cancer patients, survivors, and care givers.

## Funding

This work was supported by JSPS KAKENHI [grant number 167100000384].

## Conflict of interest

None.
